# The Influence of Seasonal Frugivory on Nutrient and Energy Intake in Wild Western Gorillas

**DOI:** 10.1371/journal.pone.0129254

**Published:** 2015-07-08

**Authors:** Shelly Masi, Roger Mundry, Sylvia Ortmann, Chloé Cipolletta, Luigi Boitani, Martha M. Robbins

**Affiliations:** 1 Max Planck Institute for Evolutionary Anthropology, D-04103, Leipzig, Germany; 2 RG Evolutionary Ecology, Leibniz-Institute for Zoo and Wildlife Research, D-10315, Berlin, Germany; 3 WCS Nyungwe Project, WSC Rwanda Program, Kigali, Rwanda; 4 Department of Animal and Human Biology, University of Rome “La Sapienza”, 00185, Rome, Italy; Curtin University, AUSTRALIA

## Abstract

The daily energy requirements of animals are determined by a combination of physical and physiological factors, but food availability may challenge the capacity to meet nutritional needs. Western gorillas (*Gorilla gorilla*) are an interesting model for investigating this topic because they are folivore-frugivores that adjust their diet and activities to seasonal variation in fruit availability. Observations of one habituated group of western gorillas in Bai-Hokou, Central African Republic (December 2004-December 2005) were used to examine seasonal variation in diet quality and nutritional intake. We tested if during the high fruit season the food consumed by western gorillas was higher in *quality* (higher in energy, sugar, fat but lower in fibre and antifeedants) than during the low fruit season. Food consumed during the high fruit season was higher in digestible energy, but not any other macronutrients. Second, we investigated whether the gorillas increased their daily *intake* of carbohydrates, metabolizable energy (KCal/g OM), or other nutrients during the high fruit season. Intake of dry matter, fibers, fat, protein and the majority of minerals and phenols decreased with increased frugivory and there was some indication of seasonal variation in intake of energy (KCal/g OM), tannins, protein/fiber ratio, and iron. Intake of non-structural carbohydrates and sugars was not influenced by fruit availability. Gorillas are probably able to extract large quantities of energy via fermentation since they rely on proteinaceous leaves during the low fruit season. Macronutrients and micronutrients, but not digestible energy, may be limited for them during times of low fruit availability because they are hind-gut fermenters. We discuss the advantages of seasonal frugivores having large dietary breath and flexibility, significant characteristics to consider in the conservation strategies of endangered species.

## Introduction

The capacity of animals to meet their nutritional and energetic needs can be challenged by seasonal fluctuations in food availability. In seasonal environments, animals are often confronted with nutritional bottlenecks during times of lower food availability [1]. The majority of primates inhabit tropical forests that can be characterized by intra-annual fluctuations in fruit availability [2]. Primates typically change their diet and activity budget in relation to seasonal variation. For example, they may minimize their energy expenditure when food is less available or increase their ranging patterns when key food resources like fruit are more abundant (e.g., [3–6]). Unraveling the relationship among the quality of dietary items consumed and dietary intake helps us to understand how animals cope with seasonally changing environments. For great apes, studies on energy budgets provide insights into how environmental challenges have led to adaptations of common ancestors with humans [7] and can help in assessing the resilience of endangered species to environmental changes (e.g., [8–12]). The aim of this study was to investigate seasonal variation in daily energy and nutrient intake in a wild great ape, the western gorilla (*Gorilla gorilla*), that shows large seasonal dietary variability as a consequence of changes in fruit availability (e.g., [13,14, 6, 15]).

The quality of available resources affects food choice and thus it may have consequences on energy budgets [16]. Such energetic effects have been shown even in species that show relatively little variation in food availability [17]. Dietary quality is normally assessed in relation to the content of energy, easily digested macronutrients (such as sugars, proteins and lipids) and micronutrients. In contrast, fibres and secondary compounds are generally inversely related to food digestibility and quality. While some secondary metabolites may be beneficial for health if ingested in small amounts (e.g., conferring medicinal properties), others are detrimental (e.g., antifeedants like tannins; e.g., [18–22]). Among plant food items, ripe fruits are considered of higher quality since they are generally richer in readily available energy (soluble sugars) and lower in fibre and antifeedants compared to leaves and herbs (e.g., [23–24], although exceptions to this generalization exist (e.g., [25–27]). However, fruit may be a costly resource to obtain because it is a contestable resource and fruit trees are typically more patchily and widely distributed than herbs and leaves [28–31]. Despite these generalisations concerning the quality of different food types, the optimal quality of diet is species-specific because of the high variability of physiological adaptations for any particular mammal species (e.g., tolerance and neutralization of antifeedants, fermentation abilities, gut flora). For non-ruminant herbivores food digestibility is further limited by body size due to factors including the amount of food intake, the capacity of the digestive tract, and gut passage rate (e.g., [24, 32–36]).

Some frugivorous primates have a much higher quality diet (macronutrients and energy) during times of high fruit abundance (e.g., [4, 37–40]) and are able to optimize their intake of antifeedants and micronutrients throughout the year [17]. However, in some cases, daily energy intake does not increase when fruit consumption increases, despite having a higher quality diet (chimpanzees and mountain gorillas; [4, 39]). Even though the caloric intake does not increase per day, the energy intake per hour is higher during high fruit consumption months in Bwindi gorillas [41].

Western gorillas are an interesting model for investigating dietary quality and intake because they are folivore-frugivores that adjust their diet and activities to seasonal variation in fruit availability. During seasons of high frugivory, western gorillas reduce the time spent feeding, but increase the time spent travelling and the distance travelled per day, presumably because they need to travel further to locate dispersed fruit trees and obtain a higher quality diet [6, 42–47]. It remains an open question whether these changes are due to the higher energy intake from a more frugivorous diet or because of some other reasons. In contrast, during periods of fruit scarcity, western gorillas rely more on leaves, fibrous fruit and herbaceous plants that are surprisingly high in protein and non-structural carbohydrates [15, 48–50]. Thus, energy intake may be potentially limited during times of low fruit availability, as observed for other primates [16, 51–52]. To elucidate this, we first tested predictions concerning the nutritional content of food items. Specifically, we predicted that leaves consumed by gorillas have higher amounts of fibre, protein, and condensed tannins than fruit, which we expected to have higher amounts of non-structural carbohydrates, sugar and energy than leaves. Secondly, we predicted that food items consumed by gorillas during the high fruit season are higher in *quality*, by having higher concentrations of energy, sugar and fat and lower amounts of fibre and condensed tannins per gram of dry matter compared to foods consumed during the low fruit season. Next, based on support for those predictions, we predicted a decrease in daily *intake* of total dry matter, fibre, secondary compounds and an increase in sugar, non-structural carbohydrates, and metabolizable energy intake (KCal/g OM) during the high fruit period to compensate for longer daily travel distances needed to locate dispersed fruit trees. No seasonal difference was expected in the protein content of food items consumed nor the quantity of protein consumed by the gorillas due to the asynchronous seasonal consumption of termites and young leaves in western gorillas that likely provide a year-round balance of protein intake [6, 47, 53–54].

## Methods

Permits and approvals for field work and biological sampling were obtained from the Ministries of Education and Water and Forests, Fishing and Hunting of the Central African Republic. This research adhered to ethics, protocols and legal requirements of the country. The study was carried out at Bai-Hokou (2° 50’ N, 16° 28’ E) located in the Dzanga sector of the Dzanga-Ndoki National Park (1222km²), Central African Republic. The area is characterized by marked seasonal variation in rainfall (drier season from December to February/March with rainfall<80mm per month; mean annual rainfall: 1700mm). Data collection on one habituated western gorilla group, the Makumba group (one silverback, four adult females, one subadult male, three juveniles and four infants for the entire study) was carried out by S.M. from December 2004 to April 2005, and from June 2005 to December 2005. For more detailed information see Masi et al. [6].

### Behavioral observations and intake rate

Data on activity and food consumed were collected during half-days and occasionally full-days of observations (N_days_ = 237, average h_obs._/day = 5) between 7:00 a.m. to 5:15 p.m. through focal animal sampling [55] of the adults, the subadult male and the juveniles. Only focal sessions longer than 15 minutes were used in the analyses. At 5-minute instantaneous intervals the activity of the focal animal was recorded (N _scans_ = 5484). Activities were classified following Masi et al. [6; 54] into four main categories: feeding (including foraging, food processing, chewing), traveling (walking, running, climbing, not related directly with foraging), resting, and social/other.

We aimed to collect at least five food intake sessions of five minute duration for each food item (combination of food type and species) per individual, randomized across the duration of feeding bouts (beginning, middle and end) to account for differential intake rate due to satiety. The amount of food ingested during a 5 minutes period (food intake rate) was recorded opportunistically either on the focal animal, when he/she was feeding for long time on one same food species, or on any other individual in view when the focal animal was engaged in long-lasting non-feeding activities such as playing or resting. During the 5 minute periods, the number of items ingested was counted for fruit, seeds, and stems, except for very large fruits (e.g., *Annonidium* sp., *Myriantus* sp.) for which the number of pulpy seeds or arils ingested was counted. This was necessary since the large size of such fruits (basketball and volleyball size respectively) typically resulted in one fruit being consumed by several individuals. For mature leaves, young leaves and termites, the numbers of handfuls ingested were counted. Bark and flowers, accounting for 1.7% and 0.7% respectively of total group feeding time, were excluded from the analysis since too few measures of intake rate and biomass ingested were made per age/sex classes. For food items that took more than five minutes to consume (e.g., fruit of large size such as *Triculia* sp.), the duration of eating a whole item was recorded. For foods for which single items were ingested in a few seconds (e.g., herb stems of *Aframomum* spp.) the number of the items consumed per unit time taken to consume them was recorded (e.g., two stems of *Aframomum* sp. in three seconds). When the intake rate (items per minute) of a given food item was not obtained for an individual, for example for the silverback, the average value for adult females was used and vice versa. Intake rates were not obtained for 17% of important food items (defined as foods consumed for more than 1% of total monthly feeding time of this study, following [28]). However, if leaf are excluded, which have less interspecies variation in shapes and size, and thus in intake rate, the missing values decreased to 10%. To estimate the missing intake rates, we used the average intake rate of a similar food type. Specifically, for unknown fruit intake rates, we used the average intake rate of similarly shaped fruits: pulpy fruit species were classified in small, medium-round, medium-long, and large based on their length and width.

### Food availability

New leaf, flower and fruit production of ten individual trees of 32 important species for the western gorilla (Bai Hokou long term data) was monitored monthly along permanent transects (e.g., [56]). We recorded the percent abundance of ripe fruit in the crown and on the ground for each tree, scoring between zero and seven (0 = 0%, 1 = 1–15%, 2 = 16–29%, 3 = 30–43%, 4 = 44–57%, 5 = 58–71%, 6 = 72–86% and 7 = 87–100% of crown and/or ground covered; modified from [57]); such scores were assigned based on the maximum crop size of each species in relation to its fruit size (e.g. a species with large fruits produces a relative smaller crop size in comparison to species with small fruits). The mean DBH (diameter at breast high) and the density of tree species were calculated from 10m^2^ plots placed every 20m along 340m long transects, which were placed systematically in 59 500x500m quadrants in the group’s home range (total plot area sampled = 9.9 ha). Finally, a monthly score of fruit abundance was calculated using a fruit availability index (FAI) calculated for each tree species as the product of the mean DBH from trees sampled in the vegetation transects, the density in 12.60 km^2^ of the group’s home range, and the mean monthly abundance score value from the phenology [40].

### Food sampling and biomass estimation

Based on the Bai Hokou long term data, the eighteen most commonly consumed species of fruit, nine leaves and young leaves, seven herbs, one bark, and one insect (termites, *Cubitermes* sp.) were collected for nutritional analysis. To account for intraspecific variation in nutritional content [25], food samples were collected in multiple subsamples from the same individual trees or plants consumed by the gorillas. Food samples were processed mimicking gorilla feeding behaviour and the parts were cut into small pieces, weighed and dried in a portable camping oven below 30–40°C. Mouldy or fermented samples were discarded. Dried samples were weighed, sealed in double plastic zip-lock bags and stored with silica gel in sealed anti-moisture containers. Termites were killed with ice immediately after harvesting to allow fast death and thus to avoid chemical alteration caused by sun or decomposition caused by slower death (bad smell occurred in these cases).

To estimate the biomass of food items, the size, wet weight and seed/pulp ratio (where applicable) were measured from 4 to 109 specimens per food item (from 46 fruit species, nine most commonly consumed species of herbs (Bai Hokou long term data), three leaves and young leaves and the termite species). For leaves and young leaves, the average weight was calculated by weighing at least 10 handfuls per species. For termites, the average wet weight of handfuls was estimated from at least 10 trials of breaking a termite mount in the upper, central and lower part, and mimicking gorilla harvesting technique. Due to high variation in diameter and length of herb pith/stem, the total stem length, the circumference, and length of the part consumed by gorillas were measured. Due to high variation in the weight of fruits, ranging from a few grams to Kg, fruit species were further classified based on the weight of the part eaten: small (< 16 g), medium (16 g = weight = 200 g), and large (> 200 g). For plant species not measured because their consumption by gorillas was low or because they had a short fruiting season (accounting for 12% of total feeding scans on important food when excluding leaves) the average weight of a similar food type or fruit category was used (as with food intake rates).

### Lab chemical analysis

Macronutrient composition of each food was analysed at the lab of the Leibniz Institute for Zoo and Wildlife Research by S.O. Before the analysis, all dried samples were grounded with an IKA A 11 Basic mill (IKA-Werke GmbH & Co. KG, 79219 Staufen, Germany) to a particle size of about 1mm. Dry matter content was obtained by drying part of the sample at 105°C overnight. Standard techniques were used for protein (Dumas combustion), fat (petroleum ether extraction, Soxhlett), and energy (bomb calorimetry). For analyses of carbohydrates (sucrose, d-glucose, d-fructose, starch) we used enzymatic tests, commercial kits from r-biopharm (R-Biopharm AG, 64297 Darmstadt, Germany) in which standard solutions were included. Additionally, a lab standard always was run in all nutrient analyses to check for reproducibility and accuracy of the tests. Ash was determined by burning dried samples in a muffle furnace for at least two hours at 550°C, after which only inorganic matter remained and the amount of ash was obtained by difference in weights. Neutral-detergent fiber (NDF) and acid detergent fiber (ADF), consisting of the three and two main structural carbohydrates of plant cell wall respectively (hemicellulose—only in NDF, cellulose and lignin), were calculated performing Detergent Fiber Analysis following van Soest [19] (for detailed results see S1 Table). For mineral analysis (Cr, Co, Mg, Fe, Ca, Cu, Na, K) samples were microwave digested and analyzed by AAS (Atom-Absorption-Spectroscopy). Total phenolics were determined according to Makkar et al. [58] (see S2 Table). Total tannins were estimated from total phenolics extracts and determination of condensed tannins followed Porter et al. [59] (see S3 Table).

Total nonstructural carbohydrates (TNC) or digestible carbohydrates were then calculated by subtraction following Conklin-Brittain et al. [60]: %TNC = 100 − (%Lipid + %Crude Protein + %Tot Ash + %NDF). Total dry matter correction coefficient (DM c.c.) was calculated as following for each sample of a given food item “*a*”: Total DM c.c. = Field DM c.c. * Final DM c.c = (g Field DM/g Field Wet matter) * (g Lab DM/g Field DM). Average grams of DM per part eaten of a given food item “*a*” (g DM _food *“a”*_) consumed by gorillas was calculated as following: g DM _food *“a”*_ = (g Fresh _food *“a”*_)*(Total DM c.c. _food *“a”*_) where *g Fresh Food “a”* is the weight in grams of the part eaten by gorillas for the food item “a”. The grams of organic matter (OM) per part eaten of a food item “*a*” (g OM _food *“a”*_) was determined as following: g OM food “*a*” = (g DM food “*a*”) * [1 − (Total Ash/g DM)].

The average value of the total correction coefficient of each food/fruit type was used to estimate the dry matter of the plant parts consumed but not analysed nutritionally. For this purpose, fruits were divided in pulpy and fibrous species with average total correction coefficient of 0.25±0.09 and 0.32±011 respectively.

### Energy intake

We calculated digestible energy of foods from the contribution of each macronutrient because the method by bomb calorimetric leads to an overestimation of energy values that also includes indigestible part plants (Table 1). To calculate energy content from plant parts consumed we used standard conversion factors: for carbohydrate (TNC) 4KCal/g, for crude protein 4KCal/g and for lipid 9KCal/g [61]. Following Conklin-Brittain et al. [60] we included a fourth conversion factor that takes into account the energy obtained by NDF fermentation. We used a mean digestion coefficient of 0.449 obtained from captive western gorillas fed a highly fibrous diet [62]. Thus the final physiological conversion factor used for NDF was: 3KCal/g * 0.449 = 1.347KCal/g. Finally, to calculate metabolizable energy we modified the equation used by Conklin-Brittain et al. [60] by using two correction factors. The first one, *protein correction*, consisted of subtracting the portion of protein per each food type that are not available to the animal after ingestion due to tannin or fiber effects [63–65]: the Acid-Detergent Insoluble Crude Proteins (ADCP), whose mean value per food type (stems, leaves, young leaves, fruit) was calculated from Conklin-Brittain et al. [65]. Secondly, for the *lignin correction*, which accounts for indigestible parts of fibers, the lignin content of each food was subtracted from NDF value since fiber coefficients and caloric conversions (4, 4, 9 KCal/g) are based on captive animals fed with low fiber diet, and on a mixed human diet including meat [60–61, 64–66]. The final formula used to calculate metabolizable energy (ME) in 100g of dry food was as following (modifications are highlighted in bold):

ME(KCal/100g OM)=(4*%TNC)+[4*(%Crude Protein−%ADCP)]+(9*%Lipid)+(1.347KCal/g*(%NDF−%Lignin)

Nutrient values are expressed as percentage of organic matter correction coefficient of the sample.

**Table 1:** Energy content of foods analysed, and relative time spent feeding on each food item in different seasons during this study.

Species	Family	Part	DE (KCal/g OM)	Energy Lab (KCal/g OM)	Fresh grams part eaten	%Feeding time	%Feeding in HF	% Feeding in LF
*Celtis mildbraedii*	ULMACEAE	BK	1.68	4.44	---	0.21	---	---
*Gambeia lacourtiana*	SAPOTACEAE	FR	4.05	4.90	96.6	0.65	0.49	0.81
*Klainnodoxa gabonensis*	IRVINGIACEAE	FR	2.38	4.13	83.62	0.44	---	0.75
*Haumania danckelmaniana*	MARANTHACEAE	FR	2.61	4.35	15.38	1.50	4.78	---
*Tetrapleura tetraptera*	MIMOSACEAE	FR	3.39	3.98	35.25	0.36	0.37	0.58
*Strombosia postulata*	OLACACEAE	FR	2.38	4.54	6.37	3.26	13.11	---
*Pancovia laurentii*	SAPINDACEAE	FR	3.32	4.22	3	1.27	---	2.19
*Diospyros crassiflora*	EBENACEAE	FR	3.65	4.07	169.8	0.28	0.98	---
*Desplatia dewerei*	TILIACEAE	FR	2.41	4.25	286.88	1.55	---	3.00
*Angyocalyx pynaertii*	PAPILLIONACEAE	FR	3.27	4.30	9.30	0.21	---	0.29
*Dialium pachyphylum*	CAESALPINACEAE	FR	3.47	4.20	0.09	8.32	20.71	---
*Annonidium manii*	ANNONACEAE	FR	3.37	4.19	15.49	0.21	0.49	---
*Dyospiros manii*	EBENACEAE	FR	3.63	4.28	109.08	0.54	1.72	---
*Vitex doniana*	VERBENACEAE	FR	3.29	4.64	12.7	0.36	0.49	---
*Nauclea sp*.	RUBIACEAE	FR	3.65	4.12	102.95	0.05	0.25	---
*Myrianthus arboreus*	MORACEAE	FR	3.20	3.86	1.19	---	---	---
*Duboscia macrocarpa*	TILIACEAE	FR	2.76	3.99	38.04	0.85	---	1.62
*Hexabolus crispiflorus*	ANNONACEAE	FR	3.35	4.16	9.3	---	---	---
*Cubitermes sp*.	TERMITINAE	IN	3.94	2.16	0.43	6.15	10.29	4.91
*Dioscorea sp*.	DIOSCORIACEAE	LV	2.88	4.66	4	0.85	0.37	0.92
*Whitefieldia elongata*	ACANTHACEAE	LV	2.60	4.17	4	0.16	0.12	0.06
*Tomadersia sp*.	ACANTHACEAE	LV	2.56	4.61	4	1.99	0.12	3.29
*(Dialium pachyphylum)*	CAESALPINACEAE	SEED-DUNG	3.32	4.47	0.333	2.84	3.55	0.17
*Gilbertiodendron dewevrei*	CAESALPINACEAE	SEED	3.06	4.05	27.2	1.45	4.53	---
*Eichornia crassipes*	PONTEDERIACEAE	ST	2.49	3.43	1.465	0.03	---	0.06
*Haumania danckelmaniana*	MARANTHACEAE	ST	2.09	4.23	2.67	2.25	3.06	1.15
*Palisota ambigua*	COMMELINACEAE	ST	1.85	3.75	9.69	0.10	---	0.17
*Aframonum subsericium*	ZINGIBERACEAE	ST	2.36	3.79	6.77	0.05	---	0.06
*Afromomum sulcatum*	ZINGIBERACEAE	ST	2.53	3.90	7.92	0.13	0.12	0.06
*Rynchospora or Scleria spp*.	CYPERACEAE	ST	1.70	3.97	2.78	5.04	---	7.85
*Palisota brachithyrsa*	COMMELINACEAE	ST	1.97	3.96	4.875	---	---	---
*Milletia sp*.	PAPILLIONACEAE	YLV	3.23	5.06	4.82	0.05	---	---
*Gilbertiodendron dewevrei*	CAESALPINACEAE	YLV	1.90	4.86	4.82	---	---	---
*Angyocalyx pynaertii*	PAPILLIONACEAE	YLV	3.00	5.14	4.29	2.92	0.49	4.73
*Dialium pachyphylum*	CAESALPINACEAE	YLV	2.29	4.55	5.36	7.21	---	14.38
*Celtis mildbraedii*	ULMACEAE	YLV	2.42	4.36	4.82	10.96	1.10	16.57
*Duboscia macrocarpa*	TILIACEAE	YLV	2.15	4.53	4.82	1.45	2.94	0.87

“Part” indicates the part of the plant or if it is an insect species consumed by gorillas: BK = bark, FR = fruit, IN = insect, LV = leaves, SEED-DUNG = seed ingested from coprophagy, ST = stem, YLV = young leaves. DE = digestible energy calculated with equation (6). Energy Lab = energy values from bomb calorimetric method. “% Feeding time” is the percentage of total feeding time from focal scans. “% Feeding in HF” and “in LF” is the % of feeding time on important food as ≥1% of the feeding time of each of the two seasons with HF = high fruit season and LF = low fruit season.

Digestible energy intake per plant species (DE_species_) per 5-minute scan per individual was calculated modifying Miller et al.[38]: DEspecies “a” (Kcal/min) = (Ir species “a”) * (g OM) * (ME KCal/g OM) where Ir _species “a”_ are items of a species “a” (i.e. fruit, handful leaves, stems) ingested per 5 minutes of the species “a”, g OM are the grams of organic matter of the part eaten by the gorillas of the species “a” (e.g. grams of one fruit or a handful). Metabolizable energy per gram of organic matter of food part eaten of the species “a” is indicated as ME kcal/g OM. The study group spent 29% of the feeding time consuming foods from species either not analyzed nutritionally or not identified. Given that within-family variation of energy content was small (Table 1), for these missing energetic and ash values we used the average kcal/g OM of similar food types from species belonging to the same genus or family. For fruit species for which genus/family value was not available, the average value of energy of the same fruit category–i.e. pulpy fruit (NDF<30 and ADF<20), fibrous fruit (NDF = 30 and ADF = 20), nuts and carobs—was used.

### Missing Values

Overall, we were unable to obtain data on nutritional analysis (including Total DM c.c.), intake rate and biomass (fresh weight of food) for food items that accounted for 21% of feeding time on important foods (see details in Table 2). However, for the majority of food items we lacked only one or two of these measurements (e.g., the nutritional analysis but not the intake rate and biomass). Even though the overall proportion of missing values for this study is slightly higher than in similar studies with missing information ranging from 9% to 17% of the feeding time [37–38, 60], the proportion becomes similar when leaves are excluded.

**Table 2:** Quantification of missing values for nutritional analysis, intake rate and food biomass.

	MISSING MEASUREMENTS			
% Total Feeding Scans				
On Important Food	*Nutritional Analysis*	*Intake Rate*	*Fresh Weight*	*All Three Variables*
All Food Items	28	27	29	21
Without Leaves	15	13	12	14

Important foods (defined as foods consumed for more than 1% of the total feeding) accounted for 98% of the total feeding scans from focal sampling. The table indicates also the percentages of missing values excluding leaves (mature and young leaves) since leaves vary less in shapes and size, thus in the rate of ingestion by gorillas.

### Statistical analysis

First, we tested for seasonal differences in *quality* of food of western gorillas using Mann-Whitney U tests. Following our previous study [6] we defined (a) a high fruit season from June to September 2005, which includes all months when fruit was consumed for approximately 70% of the group feeding scans and corresponds to higher fruit availability, and (b) low fruit seasons from December 2004 to April 2005 and November to December 2005.

Secondly, we tested the influence of fruit availability on the *intake* of (macro and micro) nutrients, energy and secondary compounds using two approaches. Because we investigated 16 interrelated variables we first ran a Factor Analysis (FA) with Varimax rotation to remove redundancy and alleviate issues of multiple testing. The FA was justified as indicated by the large correlations between some of the nutrient variables as well as the Kaiser-Meyer-Olkin measure of sampling adequacy (0.82) and Bartlett's test of sphericity (χ^2^ = 17976, df = 120, P<0.0001; [67]). Prior to the FA we transformed the variables in order to achieve distributions as symmetric as possible (Table 3). The FA revealed four factors with Eigenvalues in excess of one, together explaining 82% of the total variance (Table 3). The factors were F1) characterizing variables correlating with *“General Intake”* (including dry matter, phenols and major nutrients and minerals), F2) *“Energy Intake”* (including energy (KCal/g OM) and non-structural carbohydrates intake), F3) *“Condensed tannins”*, and F4) *“Protein/Fiber ratio*, *Na and Fe”* (including protein/fiber ratio and the important minerals such as Fe and Na).

**Table 3:** Results of the Principal Components Analysis of energy, nutrient and antifeedant intakes by the gorillas.

Intake Variables	Transf.	Factor1	Factor2	Factor3	Factor4
ADF	log(x)	**0.90**	0.29	0.24	-0.10
Mn	log(x)	**0.88**	0.13	0.18	0.03
Lignin	sqrt(x)	**0.84**	0.25	0.11	0.04
Fat	sqrt(x)	**0.84**	0.14	0.00	-0.07
Ca	log(x)	**0.79**	0.36	0.32	-0.10
Protein	log(x)	**0.79**	0.32	0.35	0.32
Mg	log(x)	**0.77**	0.41	0.33	0.19
Cu	sqrt(x)	**0.72**	0.48	0.28	0.32
Grams of Dry Matter	log(x)	**0.72**	0.61	0.28	0.11
NDF	log(x)	**0.65**	0.28	0.27	-0.36
Zn	log(x)	**0.59**	0.19	0.51	0.31
Total Phenols	x^1/4^	**0.56**	0.54	**0.56**	-0.15
TNC	log(x)	0.29	**0.87**	0.36	-0.17
Metabolizable Energy	log(x)	0.49	**0.69**	0.25	-0.02
Sum of Sugars	x^1/6^	0.19	**0.59**	0.15	-0.04
Total Tannins	x^1/4^	0.36	0.44	**0.78**	-0.24
Condensed Tannins	x^1/4^	0.18	0.37	**0.76**	-0.15
Protein/Fiber Ratio	x^1/3^	-0.19	-0.06	0.00	**0.86**
Fe	log(x)	0.40	-0.21	0.03	**0.76**
Na	x^1/6^	0.02	0.05	-0.18	**0.67**
Proportion Variance Explained		0.39	0.18	0.13	0.12
Eigenvalue		7.69	3.53	2.67	2.41

DM = dry matter; OM = organic matter; TNC = total non-structural carbohydrates (see methods for formula). Indicated are the loadings of the measures on the four Principal components derived, Eigenvalues and proportions of the variance explained per factor, and the transformations used for the different variables. Per nutrient variable the largest absolute loading is indicated in bold.

We then used general linear mixed models (GLMM, [68]) with Gaussian error structure to analyse the influence of fruit availability on energy, nutrient and antifeedant intake using the four factor scores derived as the responses (i.e., four separate analyses, one for each of the factor scores). We included only hours of observation in which more than 80% of the food intake values were known from nutritional analysis. As fixed effects we included the food availability index (log-transformed after adding 1), time of day, and time of day squared (to account for a potential time of peak food intake rate). Time of day was z-transformed to a mean of zero and a standard deviation of one and the squared term was also derived from the z-transformed time of day. We included subject identity as a random effect, but the immature individuals were pooled into a single subject. To account for potential random differences between individuals with regard to the effects of daytime and food availability on the food intake factors and to keep type I error rates at the nominal level of 0.05 we included the random slopes of these three fixed effects within individual into the model [69–70]. Food intake could show temporal autocorrelation unexplained by the predictors in the model, which could lead to non-independent residuals (i.e., residuals obtained for data points sampled close to one another in time being more similar than residuals of more distant data points). To avoid this violation of the assumption of such models, we included autocorrelation explicitly into the model. We did this by first running the model as described above and retrieving the residuals from it. We then, separately for each data point, averaged the residuals of all other data points from the same respective subject. The contribution of the residuals to this average was weighted by their time lag to the data point considered, with the weight function having the shape of a normal probability function with a mean of zero (i.e., larger weights for residuals closer in time). The standard deviation of the weight function was determined by maximizing the likelihood of the full model with the derived variable included as an additional fixed effect. The derived autocorrelation term was then included as an additional fixed effect into the full model. The model was fitted with Gaussian error and identity link. We checked for the assumptions of normally distributed and homogeneous residuals by visual inspection of qq-plots and residuals plotted against fitted values. With the exception of the model with factor 1 as the response these did not indicate severe violations of these assumptions (but given the large sample size of 708 feeding scan points we believe this model is not heavily distorted). The models were run in R (version 3.1.0; [71]) using the function lmer of the R package lme4 [72] with the argument REML set to FALSE to enable reliable likelihood ratio tests (see below). We determined P-values of fixed effects using likelihood ratio tests comparing the full model with a respective reduced model lacking the food availability index [70]. Bartlett's test of sphericity and the Kaiser-Meyer-Olkin measure of sampling adequacy were calculated using SPSS (version 20.0.0). Testing four response variables needs to account for multiple testing which we did using Fisher's omnibus test [73]. This test combines a number of P-values in a χ^2^-distributed variate with degrees of freedom equaling twice the number of P-values. A significant result indicates that one or several of the P-values are too small, assuming small P-values arose solely from multiple testing.

For the second approach, we also conducted tests of the individual nutrient variables because most other studies investigating this topic have not used Factor Analysis and interpreting the meaning of the factors may make comparisons with other studies challenging. With regard to the fixed and random effects included, these models were identical to those with the factor scores as response.

## Results

### Diet and food quality

**Fig 1:**
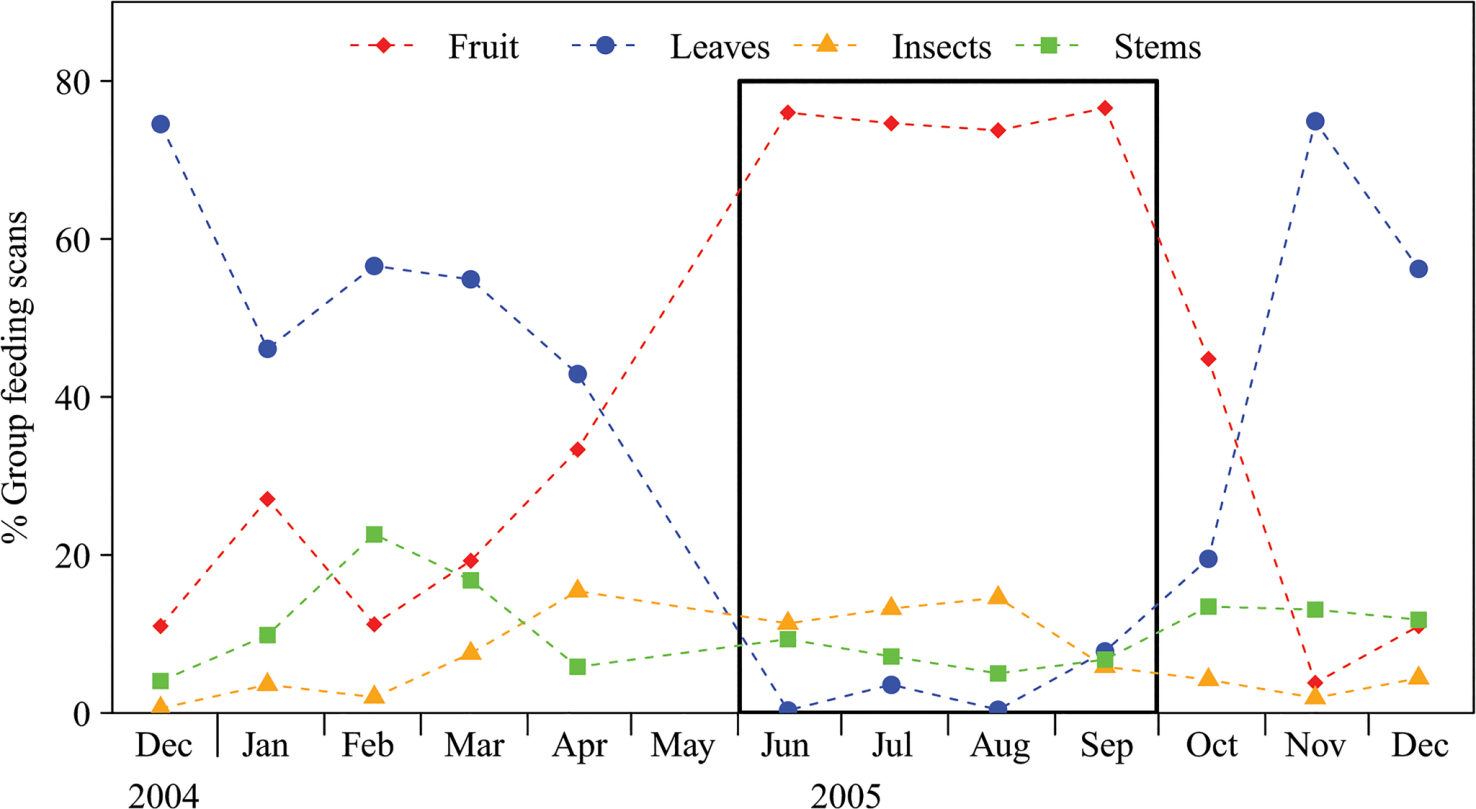
Seasonal variation in gorilla diet according to the time spend feeding on the most important food types (the bold box highlights the high-frugivory season).

During the 237 days of observations, gorillas consumed 132 food items from 98 species of plants and insects. *Important foods* (n = 68 species), defined as foods consumed for more than 1% of the total time spent feeding per month, accounted for 98.66% of the focal feeding scans. Twenty-eight of the 36 foods analysed nutritionally were important foods. The gorillas' diet changed seasonally, with fruit consumption increasing as leaf consumption decreased (Fig 1). Consumption of herbaceous stems and insects varied at a finer scale throughout the year.

As predicted, mature (LV) and young leaves (YLV) had higher protein content than fruit (FR; Mann-Whitney U test: N_YLV_ = 4, N_FR_ = 17, U = 0, P = 0.002; N_LV_ = 2, N_FR_ = 17, U = 0, P = 0.012; Table 1, see S1 Table) but less water-soluble sugars (WCS; YLV-FR: U = 2, P = 0.001; LV-FR: U = 2, P = 0.047). Young leaves also had higher NDF (U = 9, P = 0.025) and lignin content (U = 6, P = 0.012) but lower total non-structural carbohydrates than fruit (U = 3, P = 0.002). No difference was found between fruit and both young leaves and mature leaves in digestible energy (YLV-FR: U = 23, P = 0.353; LV-FR: U = 9, P = 0.345), fat (YLV-FR: U = 21, P = 0.275; LV-FR: U = 12, P = 0.573), ADF (both: U = 13, P = 0.060), or condensed tannins (or other secondary compounds, see S2 Table; YLV-FR range: U = 25–33, P = 0.462–0.965; LV-FR range: U = 10–17, P = 0.421–1; Fisher’s Omnibus test: χ^2^ = 63.78, d.f. = 12, P< 0.001).

**Fig 2:**
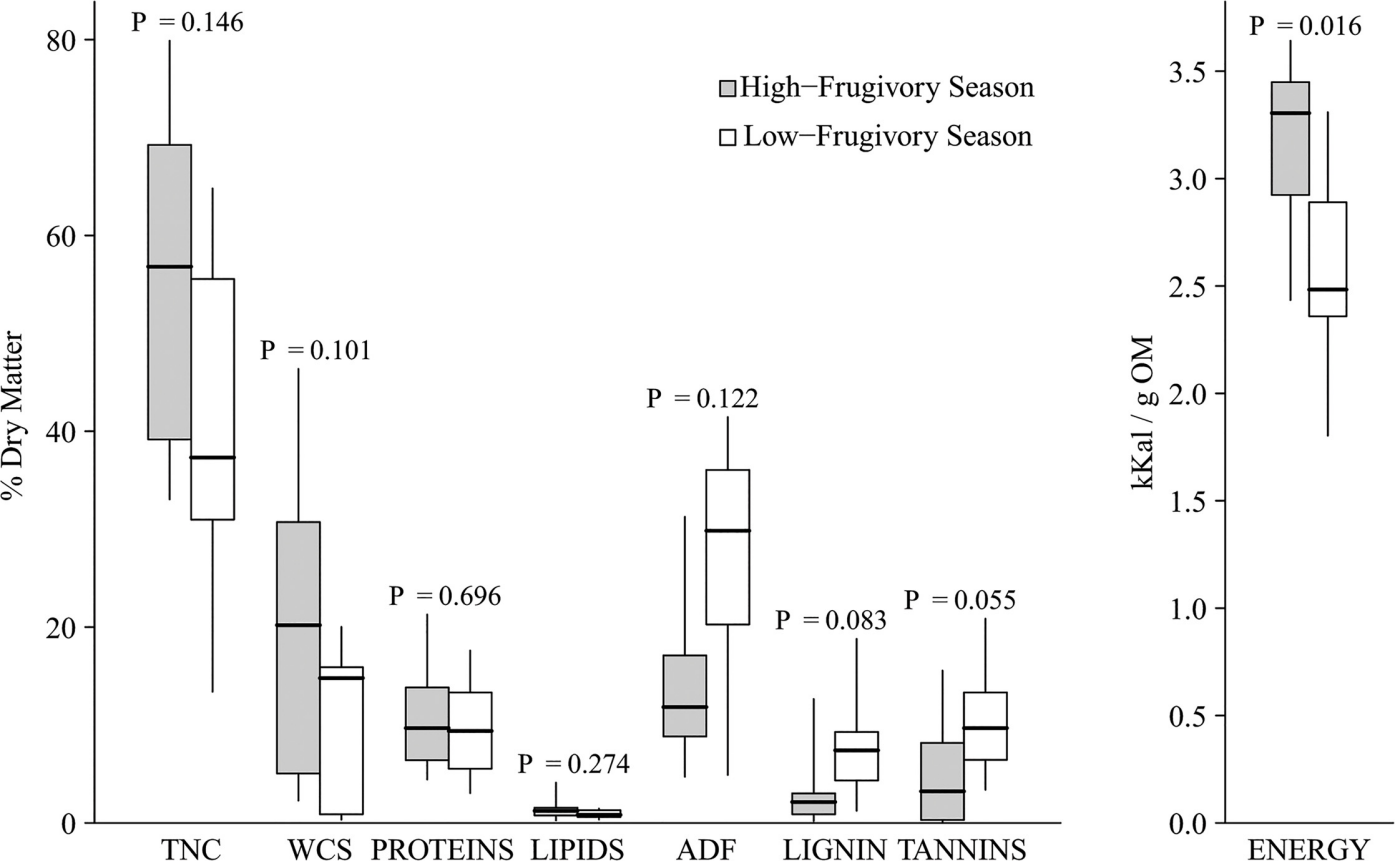
Seasonal variation in median nutritional and chemical composition of important foods (see text for definition) consumed *exclusively* in one of the two seasons (DM: dry matter; OM: organic matter; TNC: total non-structural carbohydrates; WCS: water-soluble sugars). Bold vertical lines indicate medians; boxes show the first and third quartiles, vertical lines the percentiles 2.5 and 97.5% and laying crosses denote the minimum and maximum. Numbers on the top of the graph denote the sample size.

When comparing the quality of *seasonally important foods*, that is foods consumed primarly during either the high or the low fruit seasons (defined as food with >1% of the total feeding time respectively in either season), we included only foods consumed *exclusively* in one season to be able to carry out the statistical analysis with independent samples. This led to the exclusion from the analyses of only the termites and two young leaves species. Foods consumed only during the high fruit season had higher digestible energy content (HF: 3.16±0.43Kcal/g OM) than foods consumed during the low fruit season (LF: 2.59±0.53Kcal/g OM; N_HF_ = 10, N_LF_ = 8, U = 13, P = 0.016; Fig 2). For foods consumed in both seasons we found no differences in their sugar contents (U = 21, P = 0.101), total non-structure carbohydrate (U = 23, P = 0.146), fat (U = 27, P = 0.274), protein (U = 20, P = 0.696) and ADF (U = 22, P = 0.122), or lignin (U = 20, P = 0.083; Fig 2) between seasons.

### Fruit availability and intake

Mean daily energy intake was estimated to be 5038±267 KCal/day for the silverback, 9683±225 KCal/day for lactating adult females and 8914±589 KCal/day for the immature.

**Fig 3:**
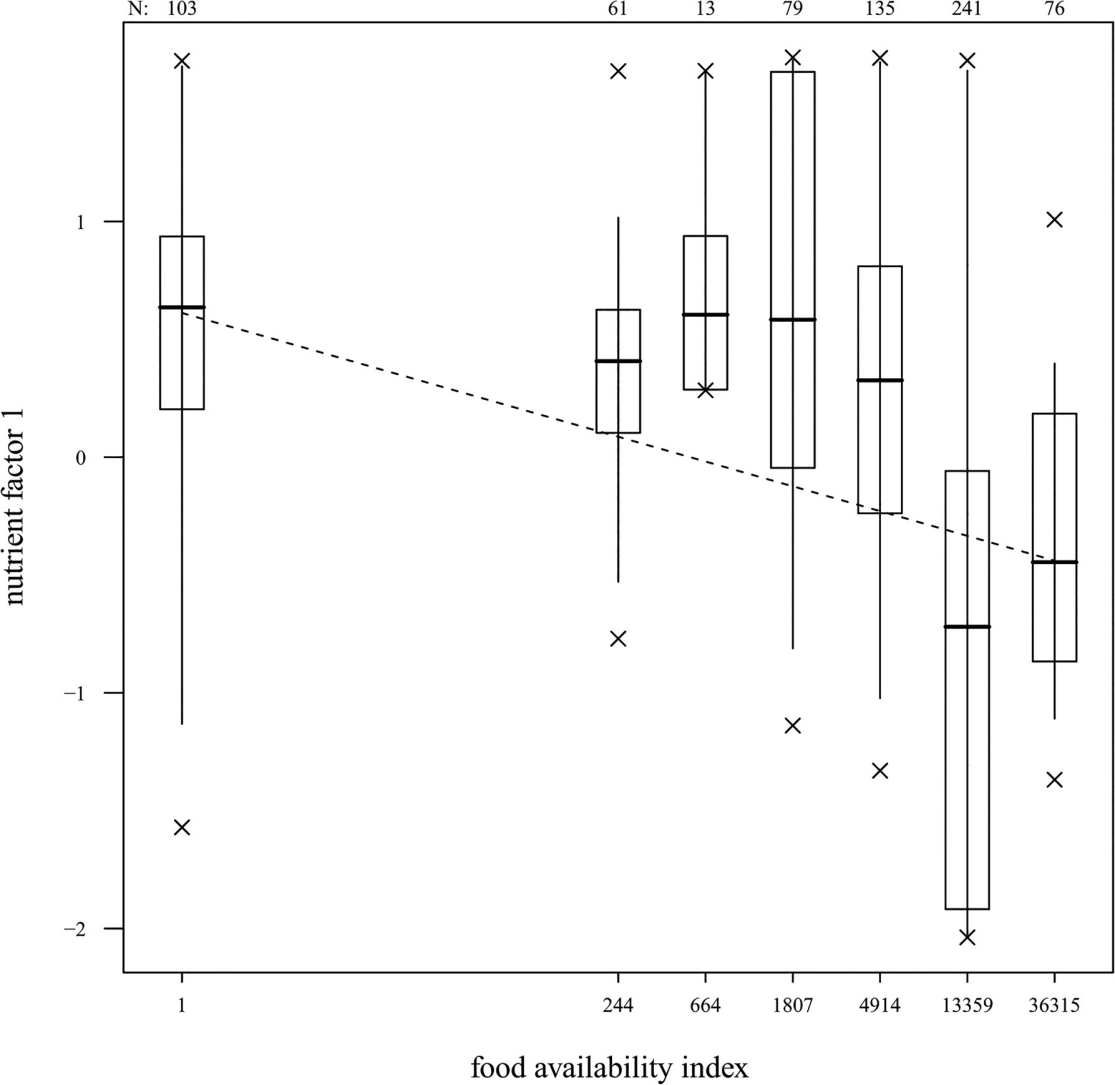
Nutrient intake (Factor 1 of PCA; see Tables 3–4a) at times of different food availability. The line shows the relation between the nutrient intake variables included in factor 1 and food availability as estimated with a GLMM (the model corresponds to that depicted in Table 5). Bold vertical lines indicate medians; boxes show the first and third quartiles, vertical lines the percentiles 2.5 and 97.5% and laying crosses denote the minimum and maximum. Numbers on the top of the graph denote the sample size.

As expected, fruit availability had a general impact on dietary intake (Fisher's omnibus test, combining four tests of the impact of fruit availability on the four nutrient factors: χ^2^ = 26.29, df = 8, P = 0.001), however the overall significance of the test was solely due to factor 1 or *“General intake”* (intake of dry matter, fibers, fat, protein and majority of minerals and phenols) which decreased with increasing fruit availably (Estimate±SE = -0.105±0.010, P<0.001; Fig 3; Table 3). A trend was revealed for the effect of fruit availability on PC2 or*“Energy Intake”* (intake of energy (KCal/g OM), sugars and non-structural carbohydrates; 0.020±0.011, P = 0.074). Contrary to what was predicted, the intake of the other two nutrient factors, including energy intake (KCal/g OM), was not significantly influenced by fruit availability (Table 4).

**Table 4:** Results of GLMMs with factor scores characterizing intakes as the response (sample size was N = 708 data points from six subjects in all models).

	Term	Estimate	SE	χ^2^	df	P
Factor 1	intercept	0.652	0.094			^(^ ^1^ ^)^
**General Intake**	FAI	-0.104	0.010	25.082	1	**<0.001**
	hour	0.085	0.069	1.333	1	0.248
	hour^2^	0.082	0.038	4.561	1	0.033
	ac.-term	0.136	0.042	9.836	1	0.002
Factor 2^(^ ^2^ ^)^	intercept	-0.194	0.110			^(^ ^1^ ^)^
**Energy Intake**	FAI	0.020	0.011	3.267	1	0.071
	hour	0.029	0.038	0.568	1	0.451
	hour^2^	0.055	0.042	1.665	1	0.197
Factor 3^(^ ^2^ ^)^	intercept	0.076	0.098			^(^ ^1^ ^)^
**Condensed Tannins**	FAI	-0.018	0.014	1.742	1	0.187
	hour	0.014	0.037	0.149	1	0.699
	hour^2^	0.012	0.041	0.086	1	0.769
Factor 4^(^ ^2^ ^)^	intercept	-0.005	0.098			^(^ ^1^ ^)^
**Protein/Fiber ratio-Na-Fe**	FAI	-0.002	0.014	0.028	1	0.867
	hour	-0.057	0.041	1.822	1	0.177
	hour^2^	-0.044	0.041	1.141	1	0.285

1: P-values for intercepts not indicated because they have no sensible interpretation.

2: The autocorrelation term was removed since it appeared insignificant (factor 2: Estimate±SE = -0.063±0.043, χ^2^ = 2.155, df = 1, P = 0.142; factor 3: 0.041±0.036, χ^2^ = 1.292, df = 1, P = 0.256) or negative, presumably an artifact of this factor having a slightly bimodal distribution and most individuals showing large values only occasionally.

When considering results of the multiple regression analyses with regard to the individual nutrient variables, we found that all 12 variables loading most strongly on *“General intake”* (factor 2 in Table 3) had significantly lower values of intake when fruit availability was greater (Table 5). Three of the other eight variables (loading most strongly on factor 2, 3, or 4), namely Metabolizable Energy, Total Tannins, and Fe, decreased with increasing fruit availability. The intake of Protein/Fibre ratio, increased with increasing fruit availability (Table 5).

**Table 5:** Results of GLMMs with the individual nutrient variables as the response analysed one at a time, showing the influence of fruit availability index (FAI) on all intakes.

Intake variable	ac^(^ ^1^ ^)^	Estimate	SE	χ^2^	df	P	PC^(^ ^2^ ^)^
**ADF**	-	-0.127	0.013	25.135	1	**<0.001**	**1**
**Mn**	-	-0.127	0.018	22.673	1	**<0.001**	**1**
**Lignin**	+	-0.157	0.014	27.719	1	**<0.001**	**1**
**Fat**	+	-0.132	0.017	23.672	1	**<0.001**	**1**
**Ca**	+	-0.116	0.020	21.734	1	**<0.001**	**1**
**Protein**	-	-0.104	0.017	14.608	1	**<0.001**	**1**
**Mg**	-	-0.094	0.018	14.148	1	**<0.001**	**1**
**Cu**	-	-0.032	0.005	15.574	1	**<0.001**	**1**
**Grams of Dry Matter**	-	-0.067	0.011	16.681	1	**<0.001**	**1**
**NDF**	-	-0.116	0.023	14.297	1	**<0.001**	**1**
**Zn**	-	-0.089	0.015	13.266	1	**<0.001**	**1**
**Total Phenols**	-	-0.020	0.005	15.064	1	**<0.001**	**1**
TNC	+	-0.017	0.012	1.880	1	0.170	2
**Metabolizable Energy**	-	-0.051	0.013	11.141	1	**0.001**	**2**
Sum of Sugars	-	0.010	0.006	2.748	1	0.097	2
**Total Tannins**	-	-0.018	0.005	13.214	1	**<0.001**	**3**
Condensed Tannins	-	-0.008	0.006	1.960	1	0.162	3
Protein/Fiber Ratio	-	0.036	0.020	2.826	1	0.093	4
**Fe**	-	-0.085	0.022	9.573	1	**0.002**	4
Na	-	-0.001	0.005	0.030	1	0.862	4

The models were the same with regard to the fixed and random effects included as for the analysis of PC factor scores.

1: Autocorrelation term included (+) or excluded from the model (-) because it was non significant (P>0.1) or negative (one model only).

2: Principal Component on which the variable was loading most strongly (see Table 2).

## Discussion

### Diet and Food quality

Our results add to previous studies conducted in different environments showing that fruit typically contains higher concentrations of readily available carbohydrates, yet lower amounts of protein and fiber than leaves (e.g., [23,24,74]; for exceptions see: [25–27,41]). In addition, as found for other primates, fruit and leaves consumed seasonally by western gorillas do not differ in levels of fat and condensed tannins ([26, 27, 75]; but see [24, 74]). Contrary to our expectations, fruits did not contain more digestible energy than leaves, suggesting that gorillas are able to extract large quantity of energy also from fibrous food [62]. Similarly, we found higher levels of digestible energy in food consumed exclusively during the high fruit season, but did not find that these foods had a higher content of fat, sugar or non-structural carbohydrates. The only evidence we found of seasonal variation of nutritional quality of food was the caloric content (digestible energy) of food, which was likely the result of the cumulative effect of the different macronutrients. Such seasonal variation in food choice explains the decrease in feeding activity by the western gorillas during the high fruit season [6]. This change in the activity budget of gorillas also may explain why Bwindi mountain gorillas show higher caloric intake per hour but not higher daily energy intake during high fruit months [39, 41]. Indeed, many primate species, including western gorillas, respond to seasonal variation in fruit availability by modifying their activity budget and/or diet through reliance on alternative food resources such as young leaves and herbaceous vegetation rich in proteins (e.g., [4, 6, 15, 41, 76–78]). Thus, even though quality of leaves and fruit may vary, western gorillas appear to be able to balance the nutrient compositions of food items ingested regardless of season. In contrast, some other frugivorous primates have a much higher quality of diet in terms of both macronutrients and energy during times of high fruit abundance (e.g., golden lion tamarins: [38]; baboons: [55]; chimpanzees and orang-utans: [4, 37, 40, 52]; mountain gorillas: [39]). Fiber content of foods consumed does not vary seasonally for African apes, but the diet of Borneo orangutans includes higher levels of fiber when fruit is scarce [4, 37, 39, 52].

### Dietary Intake

The daily energy intake measured in western gorillas (5038±267 KCal/day for the silverback, 9683±225 KCal/day for lactating adult females and 8914±589 KCal/day for the immature) was similar to that of mountain gorillas [39]: silverbacks = 9202±470 KCal/day, adult females (including four lactating females and two non-pregnant or non-lactating females) = 8178±588 KCal/day, juveniles = 7124±694. Comparison between the two studies should be done cautiously due to small sample sizes in each. The larger value found for the silverback mountain gorilla may be explained partially by the larger size of this species (eastern gorillas) than the western species, and also possibly due to extrapolation of limited observation time by Rothman et al. [39] to reflect a full day estimate of caloric intake. Differences in caloric intake by adult females and immatures could be due to the proposed larger cost of reproduction and growth (e.g., later age at weaning, longer interbirth interval and age to sexual maturity) for western gorillas than mountain gorillas, since they inhabit a more seasonal and unpredictable environment [79, 80].

Some studies investigating the influence of seasonality on food intake found that energy and macronutrients (sugars, carbohydrates, and fats) may be limited during times of low fruit availability (howler monkeys: [16]; woolly monkeys: [51]; chimpanzees: [4]; orang-utans: [37, 52]; mountain gorillas: [39]). The main result of this study, validated by both statistical approaches (factor scores and individual variables), showed that the *“General Intake”* of major macro- and micronutrients (such as fibers, fat, protein and the majority of minerals), phenols and the intake of dry matter decreased with increases of both frugivory and fruit availability. Therefore, the benefit associated with a higher caloric and more digestible diet during the high fruit season (as found by this study; see “*Diet and Food quality*”) seem to occur at the detriment of a general nutrient intake (i.e., dry matter, fibers, fat, protein, and the majority of minerals) and thus nutrient diversity (but this is true also for certain secondary compounds such as phenols). Whether there was seasonal variation in the variables for reflecting energy intake (KCal/g OM), antifeedants and other nutrients varied, depending on the statistical analysis used. Based on the analysis that combined particular variables in factor scores, daily *“Energy Intake”* (intake of non-structural carbohydrates, sugars and energy—KCal/g OM), *“Condensed tannins”*, and *“Protein/Fiber ratio*, *Na and Fe”* were not found to be influenced by fruit abundance, even though “*Energy Intake”* showed a trend. Yet when each variable was analyzed independently we found that metabolizable energy, total tannins, protein/fiber ratio, and iron showed seasonal variation. The results of the individual variables should be treated with caution due to multiple testing, but it also cannot be ruled out that some seasonal effects on the individual variables were obscured by combining variables in the factor analysis. To our knowledge, no other studies investigating the influence of fruit availability on energy and nutrient intake in wild great apes have used Factor Analysis, but instead analyses have based on the individual variables. The Factor Analysis is an appropriate method because it reduces the number of dependent variables that may covary in ways that are difficult to predict and eliminates the problem of multiple-testing. However, to interpret the biological meaning of all the factors can be difficult. In contrast, the multiple regression analysis is more comparable with previous studies.

Based on these main findings, macro- and micro-nutrients are likely not limited for western gorillas during times of low fruit availability. Indeed, to fuel their nutritional needs during the low fruit season, western gorillas increased the time spent feeding and ingested greater quantities of dry matter to compensate for the lower content of digestible energy found in food consumed during this season [6]. The result of this “time maximiser” strategy is that when fruit is scarce, western gorillas likely extract the majority of their daily energy requirements through an increased ingestion of proteins, fats and fibers from leaves and bark, made possible due to their large body size and ability as hind-gut fermenters [32, 62]. This is in contrast to other apes with less possibility to ferment foods, such as orangutans, because of the high lignin level in the food [60, 81], or chimpanzees that a) usually produce wadges of ingested food (i.e., *Ficus* spp.) to reject the most fibrous part of the food, a behavior that is absent in wild gorillas, or b) discard more fibrous plant parts that are typically ingested by the western gorillas (e.g., a part of the pith of the *Aframomum* spp.) (Masi, pers. obs.). In sum, the higher quality and availability of alternative resources in the environment and the greater ability to ferment foods allow larger bodied African apes to consume a higher quality diet year-round as compared to orangutans and many other frugivorous primates.

The physiological abilities to ferment and neutralize plant toxins [32, 62] allow gorillas to over-ingest proteins from non-reproductive and fibrous plant parts, thereby implementing a protein-limited diet during periods of fruit scarcity. In fact, as predicted in terms of protein intake, the lower reliance on termites during the low fruit period (Fig 1) is likely compensated by a greater reliance on herbaceous plants and leaves that may result in a surplus of plant proteins and iron compared to what is predicted for their body size, as it has been suggested for other primates [82–83]. This apparent surplus may be obligatory to balance their protein intake throughout the year since 20% of total nitrogen concentration in wild plant parts is not available to primates [82], and even the most digestible plant proteins show lower digestibility in comparison to animal proteins [84]. Even though protein content has been suggested to affect primate biomass as well as the evolution of primate diversity [11, 85, 86], mountain gorillas do not appear to prioritize protein intake over energy intake, in contrast to humans and other primates [81, 83, 87, 88]. Western gorillas may do the same as mountain gorillas during the period of lower reliance on animal matter (termites). The abnormally high levels (with respect to human standards) of nitrites found in the urine of the study group [89] supports this hypothesis since gorillas may be physiologically adapted to excrete excess nitrogen (apes: [81, 83]; other mammals: [90, 91]). Because we obtained different results depending on the statistical approach used, we were unable to determine whether the gorillas are able to better optimize protein/fibre intake when feeding on fruit.

Sodium (Na) was the only mineral whose intake clearly did not vary across the year, highlighting that western gorillas may balance and prioritize the intake of this mineral more than other micronutrients (similarly to mountain gorillas see [26]). In contrast, the intake of Zn, Cu, Mn, Mg, and Ca varied according to fruit availability. Lastly, changes in fruit availability seem to differentially influence the intake of secondary compounds: no influence was observed on tannin intake, while total phenols and total tannins (as suggested by the analysis of individual variables) may become problematic for western gorillas when they are more folivorous (i.e., during low fruit availability). This suggests they may rely on a strategy to minimize ingestion of antifeedants in the low fruit period and/or that their tolerance to toxins may be relatively high thanks to their physiological adaptations. Further studies using both statistical approaches are needed to obtain more conclusive findings for this and other species.

As observed for the other African apes, this overall pattern of nutrient intake allows western gorillas to maintain a constant daily energy intake throughout the year despite the large seasonal variation in fruit availability [39, 60]. However, our results also suggest that there may be seasonal variation on a finer level since metabolizable energy intake (KCal/g OM) appears to increase in low fruit period, presumably made available in the herbaceous vegetation they consume and their fermentation ability. In contrast, other primate species respond to seasonal fluctuations in food availability by an “energy maximiser” strategy in which they greatly exceed the daily caloric requirements to store fat during periods of high fruit abundance and subsist on lower quality food during lean seasons (lemurs: [92]; baboons: [93]; orangutans: [37, 52]). The greater variance in seasonality and in the quality of food available in their habitats may explain the different strategies adopted by these primates, particularly the differences among the African and the Asian great apes (chimpanzees and gorillas vs. orangutans; [52, 77]. Moreover, the physiological adaptations to herbivory and the greater ability to digest fibers due to the larger body size and longer gut of great apes [32] may account for some of the differences in dietary strategies compared to the smaller primates (baboons and lemurs). Therefore, dietary flexibility and specific physiological adaptations may enable particular primate species to increase their resilience to unpredictable changes in the environment. This may be significant information in assisting the conservation strategies for endangered species.

### Why be seasonally frugivorous?

Our results of seasonal variation in dietary intake by western gorillas suggest that they may face more nutritional challenges than what would appear by investigating only the nutritional quality of food consumed seasonally or the total energy intake across the year. Results on the same study group showing that the occurrence of parasites and urinary infection rates in western gorillas also vary seasonally highlight that they may face seasonal nutritional stress that impacts their health [89]. However, since the daily energy intake (KCal/g OM) of western gorillas, as for the other African apes, does not appear to increase with frugivory, despite their increase in daily travel distance and time, we may question how they are able to balance their energy budget throughout the year (e.g., [6, 46]).

Since western gorillas are able to profit from a high quality diet (particularly from proteinaceous young leaves) even when fruit is scarce, what is the advantage of switching their diet and searching for fruit when it is available? Despite the greater energy effort needed to locate fruiting trees, different factors may make the strategy of seasonal frugivory beneficial for primates. First, fruit may be a suitable nutritional alternative in periods of the year when high quality young leaves are less available (Bai Hokou, long term data). Second, a preference for sweetness or the tendency for sweetness to elicit positive affective reactions is widespread across all primate species including gorillas [94, 95], suggesting an early evolution of fruit-eating in ancestral primates [96]. Sweetness is related to the presence of readily accessible nutrients (sugars) found generally at higher concentrations in fruit than other foods (i.e., leaves). Additionally, because of the higher digestibility of fruit, primates have more time to invest in other activities when they are able to reduce time spent feeding and the amount of organic matter ingested [6, 97, 98]. For example, during the high fruit season western gorillas increase the time spent being social (i.e., increasing playing time, [6]; even the adults, Masi, pers. obs.) and time spent travelling [6], which may enable them to search for micronutrients that are less available and consumed less during this season (i.e. Mn, Zn, Ca, Mg, Cu; Tables 3 and 4a-b). They also may be able to increase the efficiency of the ingestion of protein/fiber, which may be a strategy to decrease antifeedant intake resulting in lower levels of toxins at least for some months of the year. Third, a wider dietary breadth per year provides animals, including humans, with greater variety of nutritional and chemical substances that provides both nutritional and health benefits [35, 99]. Our study found that the quantity and diversity of macro- and micro-nutrient intake decrease when gorillas prioritize an energy-rich diet based on fruit. A similar pattern was found for phenols, which may be beneficial to maintain the gorilla health even though they are toxic in certain cases [100]. Lastly, large dietary flexibility is crucial for resilience and survival in the face of natural and anthropogenic changes in the environment.

## Supporting Information

S1 Table Macronutrient and ash content of western gorilla food indicated as percentages of dry matter. (DOC)

S2 Table Secondary compounds content of western gorilla food indicated as percentages of dry matter. (DOCX)

S3 Table Micronutrient content of western gorilla food indicated as mg/Kg of dry matter. (DOCX)
