# Supplementary material for: The Influence of Seasonal Frugivory on Nutrient and Energy Intake in Wild Western Gorillas
Source: PLoS One. 2015 Jul 8;10(7):e0129254. doi: 10.1371/journal.pone.0129254 (PMC4495928; doi:10.1371/journal.pone.0129254)
Supplement: S1 Table — (DOC) [file pone.0129254.s001.doc]

**Table S1. Macronutrient and ash content of western gorilla food indicated as percentages of dry matter.**

| **Species** | **Ba'Aka name** | **Family** | **Part** | **Ash** | **OM** | **CP** | **Starch** | **Fructose** | **Glucose** | **Sucrose** | **TNC** | **NDF** | **ADF** | **ADL** | **Cel.** | **Hemicel.** | **Fat** |
| --- | --- | --- | --- | --- | --- | --- | --- | --- | --- | --- | --- | --- | --- | --- | --- | --- | --- |
| *Celtis mildbraedii* | NGOMBE | ULMACEAE | BK | 6.0 | 94.0 | 19.3 | 1.4 | 0.1 | 0.0 | 1.7 | 9.0 | 67.7 | 47.6 | 20.0 | 27.6 | 20.1 | 1.0 |
| *Angyocalyx pynaertii* | MANJOMBE | PAPILLIONACEAE | FR | 1.9 | 98.1 | 12.2 | 17.4 | 5.3 | 4.4 | 6.2 | 63.0 | 22.7 | 4.6 | 1.1 | 3.5 | 18.0 | 0.3 |
| *Annonidium manii* | MOBEI | ANNONACEAE | FR | 15.2 | 84.8 | 9.7 | 4.0 | 12.7 | 10.4 | 2.9 | 56.1 | 17.7 | 11.7 | 2.2 | 9.4 | 6.0 | 1.3 |
| *Desplatia dewerei* | LIAMBA | TILIACEAE | FR | 4.4 | 95.6 | 8.4 | 0.6 | 3.6 | 2.2 | 10.2 | 35.0 | 50.9 | 41.8 | 9.3 | 32.5 | 9.2 | 1.3 |
| *Dialium zenkeri/pachyphylum* | MBASO | CAESALPINACEAE | FR | 2.4 | 97.6 | 4.2 | 1.9 | 19.4 | 20.0 | 8.2 | 77.0 | 15.6 | 8.3 | 1.6 | 6.7 | 7.3 | 0.7 |
| *Diospyros crassiflora* | LEMBE | EBENACEAE | FR | 2.8 | 97.2 | 5.4 | 0.1 | 14.3 | 5.6 | 22.0 | 80.7 | 10.3 | 7.7 | 0.1 | 7.6 | 2.6 | 0.8 |
| *Duboscia macrocarpa* | NGULUMA | TILIACEAE | FR | 3.2 | 96.8 | 3.2 | 0.0 | 4.8 | 8.6 | 2.2 | 53.1 | 39.2 | 31.1 | 8.2 | 22.9 | 8.1 | 1.3 |
| *Dyospiros manii* | MOLOMBO | EBENACEAE | FR | 6.0 | 94.0 | 7.2 | 0.2 | 6.0 | 3.9 | 22.5 | 71.4 | 13.1 | 10.3 | 0.6 | 9.7 | 2.8 | 2.3 |
| *Gambeya lacourtiana* | BAMBU | SAPOTACEAE | FR | 3.5 | 96.5 | 7.9 | 0.0 | 6.3 | 4.4 | 4.5 | 57.3 | 17.3 | 11.9 | 6.2 | 5.6 | 5.4 | 14.1 |
| *Haumania danckelmaniana* | DJELE | MARANTHACEAE | FR | 3.9 | 96.1 | 12.6 | 25.0 | 0.7 | 0.4 | 1.0 | 34.5 | 47.4 | 17.7 | 3.2 | 14.5 | 29.7 | 1.6 |
| *Hexabolus crispiflorus* | POTA | ANNONACEAE | FR | 5.1 | 94.9 | 8.0 | 1.4 | 16.9 | 14.6 | 14.4 | 68.8 | 18.1 | 14.1 | 2.2 | 11.9 | 4.0 |  |
| *Klainnodoxa gabonensis* | BOKOKO | IRVINGIACEAE | FR | 3.6 | 96.4 | 3.0 | 0.1 | 2.5 | 1.1 | 17.3 | 39.7 | 53.1 | 25.0 | 5.2 | 19.8 | 28.2 | 0.6 |
| *Myrianthus arboreus* | NGATA | MORACEAE | FR | 4.3 | 95.7 | 5.8 | 0.1 | 20.7 | 18.7 | 7.2 | 70.0 | 21.4 | 15.6 | 3.1 | 12.5 | 5.8 | 0.6 |
| *Nauclea sp.* | MOSSE TI NGU | RUBIACEAE | FR | 2.4 | 97.6 | 6.5 | 0.1 | 11.5 | 10.1 | 2.7 | 65.7 | 18.7 | 14.1 | 4.8 | 9.4 | 4.6 | 6.7 |
| *Pancovia laurentii* | INGOYO | SAPINDACEAE | FR | 2.7 | 97.3 | 10.4 | 15.3 | 4.8 | 4.0 | 5.2 | 65.2 | 21.1 | 6.2 | 1.8 | 4.4 | 14.9 | 0.6 |
| *Strombosia postulata* | EMBONGO | OLACACEAE | FR | 3.8 | 96.2 | 14.2 | 0.0 | 14.5 | 2.2 | 0.0 | 32.6 | 48.6 | 34.8 | 14.2 | 20.7 | 13.8 | 0.7 |
| *Tetrapleura tetraptera* | EKOMBOLO | MIMOSACEAE | FR | 3.3 | 96.7 | 7.3 | 0.0 | 2.2 | 3.7 | 47.0 | 72.8 | 16.3 | 12.0 | 4.1 | 7.9 | 4.3 | 0.2 |
| *Vitex doniana* | MONGWENGWEA | VERBENACEAE | FR | 9.5 | 90.5 | 9.6 | 0.3 | 0.2 | 17.3 | 6.1 | 57.5 | 21.9 | 15.3 | 2.1 | 13.3 | 6.5 | 1.4 |
| *Cubitermes sp.* | KUSSU | TERMITINAE | IN | 59.4 | 40.6 | 18.0 | 0.6 | 0.0 | 0.0 | 0.0 | 5.8 | 10.6 | 7.0 | 3.8 | 3.3 | 3.6 | 6.2 |
| *Dioscorea sp.* | EKULE | DIOSCORIACEAE | LV | 5.2 | 94.8 | 19.8 | 0.3 | 2.5 | 2.9 | 0.2 | 38.7 | 35.1 | 19.0 | 7.4 | 11.6 | 16.2 | 1.2 |
| *Tomadersia sp.* | INGUKA | ACANTHACEAE | LV | 5.3 | 94.7 | 17.8 | 0.6 | 0.4 | 0.6 | 0.0 | 29.3 | 46.1 | 28.6 | 9.3 | 19.3 | 17.5 | 1.5 |
| *Whitefieldia elongata* | INDOLU | ACANTHACEAE | LV | 12.9 | 87.1 | 33.9 | 0.1 | 0.2 | 0.1 | 0.4 | 9.7 | 41.9 | 23.2 | 7.7 | 15.5 | 18.7 | 1.6 |
| *Gilbertiodendron dewevrei* | MALAPA | CAESALPINACEAE | SEED | 1.7 | 98.3 | 6.1 | 25.4 | 0.2 | 0.2 | 4.5 | 62.8 | 29.2 | 3.9 | 2.4 | 1.5 | 25.3 | 0.1 |
| *(Dialium pachyphylum)* | PURU_MBASO | CAESALPINACEAE | SEED-DUNG | 2.9 | 97.1 | 21.7 | 0.3 | 0.4 | 0.3 | 2.1 | 40.5 | 33.2 | 12.0 | 0.6 | 11.4 | 21.1 | 4.6 |
| *Aframonum sp.* | INJOMBO | ZINGIBERACEAE | ST | 14.3 | 85.7 | 14.2 | 0.4 | 3.3 | 2.8 | 3.0 | 21.9 | 47.1 | 27.3 | 2.5 | 24.8 | 19.9 | 2.4 |
| *Aframonum subsericium* | INJOKOKO | ZINGIBERACEAE | ST | 13.9 | 86.1 | 9.6 | 0.7 | 2.4 | 2.3 | 4.5 | 23.6 | 51.2 | 32.2 | 3.1 | 29.1 | 19.1 | 1.7 |
| *Eichornia crassipes* | CONGWASSIKA | PONTEDERIACEAE | ST | 26.7 | 73.3 | 10.9 | 4.0 | 2.2 | 1.6 | 0.7 |  | 41.3 | 25.2 | 2.0 | 23.2 | 16.1 | 0.8 |
| *Haumania danckelmaniana* | DJELE | MARANTHACEAE | ST | 10.3 | 89.7 | 39.3 | 0.1 | 0.1 | 0.0 | 0.0 | 0.0 | 49.5 | 24.8 | 8.0 | 16.8 | 24.7 | 2.7 |
| *Palisota ambigua* | DOTO | COMMELINACEAE | ST | 12.8 | 87.2 | 8.8 | 0.0 | 0.5 | 0.5 | 0.2 | 13.3 | 64.8 | 44.6 | 5.0 | 39.6 | 20.2 | 0.3 |
| *Palisota brachithyrsa* | MANGABO | COMMELINACEAE | ST | 8.3 | 91.7 | 7.9 | 1.5 | 4.3 | 1.3 | 0.9 | 19.1 | 65.2 | 46.0 | 5.3 | 40.7 | 19.2 | 0.6 |
| *Scleria sp.* | KYEYE | CYPERACEAE | ST | 9.6 | 90.4 | 6.3 | 1.4 | 0.1 | 0.1 | 0.3 | 10.0 | 73.0 | 39.9 | 6.6 | 33.2 | 33.1 | 1.0 |
| *Angyocalyx pynaertii* | MANJOMBE | PAPILLIONACEAE | YLV | 4.7 | 95.3 | 28.3 | 0.6 | 0.0 | 0.0 | 0.7 | 35.7 | 29.6 | 16.7 | 9.1 | 7.6 | 13.0 | 1.6 |
| *Celtis mildbraedii* | NGOMBE | ULMACEAE | YLV | 7.6 | 92.4 | 21.9 | 3.2 | 1.9 | 1.7 | 1.0 | 15.5 | 51.9 | 22.7 | 9.2 | 13.5 | 29.2 | 3.1 |
| **Species** | **Ba'Aka name** | **Family** | **Part** | **Ash** | **OM** | **CP** | **Starch** | **Fructose** | **Glucose** | **Sucrose** | **TNC** | **NDF** | **ADF** | **ADL** | **Cel.** | **Hemicel.** | **Fat** |
| *Dialium pachyphylum* | MBASO | CAESALPINACEAE | YLV | 2.4 | 97.6 | 16.8 | 0.0 | 0.1 | 0.0 | 0.2 | 31.5 | 48.6 | 34.8 | 20.8 | 14.0 | 13.8 | 0.6 |
| *Duboscia macrocarpa* | NGULUMA | TILIACEAE | YLV | 7.8 | 92.2 | 17.8 | 2.0 | 1.1 | 1.0 | 0.6 | 13.6 | 58.9 | 32.0 | 9.0 | 23.0 | 26.9 | 1.8 |
| *Gilbertiodendron dewevrei* | MALAPA | CAESALPINACEAE | YLV | 3.2 | 96.8 | 15.2 | 0.0 | 0.1 | 0.1 | 0.3 | 19.0 | 61.5 | 50.8 | 24.9 | 25.9 | 10.7 | 1.1 |
| *Milletia sp.* | INGANDA | PAPILLIONACEAE | YLV | 5.9 | 94.1 | 40.4 | 0.1 | 0.0 | 0.0 | 0.4 | 22.1 | 27.8 | 13.2 | 3.7 | 9.5 | 14.6 | 3.7 |
|  |  |  |  |  |  |  |  |  |  |  |  |  |  |  |  |  |  |

OM = organic matter, CP = crude protein, TNC = total non-structural carbohydrates, NDF = neutral detergent fiber, ADF = acid detergent fiber, ADL = acid detergent lignin, Cel. = cellulose, Hemicel. = hemicellulose. “Part” indicates the part of the plant (or insect species) consumed by gorillas: BK = bark, FR = fruit, IN = insect, LV = leaves, SEED-DUNG = seed ingested from coprophagy, ST = stem/pith, YLV = young leaves.
