# Supplementary material for: The Influence of Seasonal Frugivory on Nutrient and Energy Intake in Wild Western Gorillas
Source: PLoS One. 2015 Jul 8;10(7):e0129254. doi: 10.1371/journal.pone.0129254 (PMC4495928; doi:10.1371/journal.pone.0129254)
Supplement: S2 Table — (DOCX) [file pone.0129254.s002.docx]

**Table S2. Secondary compounds content of western gorilla food indicated as percentages of dry matter.**

| **Species** | **Ba'Aka name** | **Family** | **Part** | **TP** | **TT** | **CT** |
| --- | --- | --- | --- | --- | --- | --- |
| *Celtis mildbraedii* | NGOMBE | ULMACEAE | BK | 1.8 | 0.6 | 0.0 |
| *Angyocalyx pynaertii* | MANJOMBE | PAPILLIONACEAE | FR | 1.2 | 0.5 | 1.0 |
| *Annonidium manii* | MOBEI | ANNONACEAE | FR | 1.7 | 1.0 | 0.4 |
| *Desplatia dewerei* | LIAMBA | TILIACEAE | FR | 4.1 | 2.9 | 2.8 |
| *Dialium zenkeri/pachyphylum* | MBASO | CAESALPINACEAE | FR | 0.9 | 0.7 | 0.3 |
| *Diospyros crassiflora* | LEMBE | EBENACEAE | FR | 9.0 | 6.8 | 0.1 |
| *Duboscia macrocarpa* | NGULUMA | TILIACEAE | FR | 0.8 | 0.6 | 1.3 |
| *Dyospiros manii* | MOLOMBO | EBENACEAE | FR | 10.4 | 2.2 | 10.7 |
| *Gambeya lacourtiana* | BAMBU | SAPOTACEAE | FR | 5.8 | 5.5 | 7.5 |
| *Haumania danckelmaniana* | DJELE | MARANTHACEAE | FR | 4.2 | 3.1 | 4.4 |
| *Hexabolus crispiflorus* | POTA | ANNONACEAE | FR |  |  |  |
| *Klainnodoxa gabonensis* | BOKOKO | IRVINGIACEAE | FR | 6.3 | 5.5 | 3.5 |
| *Myrianthus arboreus* | NGATA | MORACEAE | FR | 0.8 | 0.0 | 0.7 |
| *Nauclea sp.* | MOSSE TI NGU | RUBIACEAE | FR | 1.2 | 0.9 | 1.9 |
| *Pancovia laurentii* | INGOYO | SAPINDACEAE | FR | 0.9 | 0.7 | 0.9 |
| *Strombosia postulata* | EMBONGO | OLACACEAE | FR | 4.1 | 3.1 | 3.8 |
| *Tetrapleura tetraptera* | EKOMBOLO | MIMOSACEAE | FR | 5.9 | 4.9 | 2.9 |
| *Vitex doniana* | MONGWENGWEA | VERBENACEAE | FR | 5.1 | 2.6 | 0.1 |
| *Cubitermes sp.* | KUSSU | TERMITINAE | IN |  |  |  |
| *Dioscorea sp.* | EKULE | DIOSCORIACEAE | LV | 17.5 | 15.9 | 17.0 |
| *Tomadersia sp.* | INGUKA | ACANTHACEAE | LV | 3.0 | 1.2 | 0.0 |
| *Whitefieldia elongata* | INDOLU | ACANTHACEAE | LV | 0.5 | 0.3 | 0.0 |
| *Gilbertiodendron dewevrei* | MALAPA | CAESALPINACEAE | SEED | 3.8 | 2.9 | 2.7 |
| *(Dialium pachyphylum)* | PURU_MBASO | CAESALPINACEAE | SEED-DUNG | 6.1 | 5.6 | 9.4 |
| *Aframonum sp.* | INJOMBO | ZINGIBERACEAE | ST |  |  |  |
| *Aframonum subsericium* | INJOKOKO | ZINGIBERACEAE | ST | 2.0 | 1.8 | 2.3 |
| *Eichornia crassipes* | CONGWASSIKA | PONTEDERIACEAE | ST | 0.5 | 0.3 | 0.3 |
| *Haumania danckelmaniana* | DJELE | MARANTHACEAE | ST | 3.0 | 1.2 | 0.0 |
| *Palisota ambigua* | DOTO | COMMELINACEAE | ST | 0.6 | 0.4 | 0.3 |
| *Palisota brachithyrsa* | MANGABO | COMMELINACEAE | ST |  |  |  |
| *Scleria sp.* | KYEYE | CYPERACEAE | ST | 0.3 | 0.1 | 0.0 |
| *Angyocalyx pynaertii* | MANJOMBE | PAPILLIONACEAE | YLV | 6.2 | 4.9 | 8.2 |
| *Celtis mildbraedii* | NGOMBE | ULMACEAE | YLV | 2.9 | 0.3 | 0.0 |
| *Dialium pachyphylum* | MBASO | CAESALPINACEAE | YLV | 3.6 | 2.6 | 0.2 |
| *Duboscia macrocarpa* | NGULUMA | TILIACEAE | YLV | 1.9 | 1.0 | 1.2 |
| *Gilbertiodendron dewevrei* | MALAPA | CAESALPINACEAE | YLV | 7.0 | 5.7 | 12.3 |
| *Milletia sp.* | INGANDA | PAPILLIONACEAE | YLV | 3.2 | 1.4 | 0.9 |
|  |  |  |  |  |  |  |

TP = total phenolics, TT = total tannins, CT = condensed tannins. See Appendix 1 for codes of column “Part”.
