# Supplementary material for: The Influence of Seasonal Frugivory on Nutrient and Energy Intake in Wild Western Gorillas
Source: PLoS One. 2015 Jul 8;10(7):e0129254. doi: 10.1371/journal.pone.0129254 (PMC4495928; doi:10.1371/journal.pone.0129254)
Supplement: S3 Table — (DOCX) [file pone.0129254.s003.docx]

**Table S3.** **Micronutrient content of western gorilla food indicated as mg/Kg of dry matter.**

| **Species** | **Ba'Aka name** | **Family** | **Part** | **Ca** | **Cu** | **Fe** | **Mg** | **Mn** | **Na** | **Zn** |
| --- | --- | --- | --- | --- | --- | --- | --- | --- | --- | --- |
| *Celtis mildbraedii* | NGOMBE | ULMACEAE | BK | 27764,1 | 4,4 | 9,4 | 690,4 | 268,5 | 8,5 | 6,2 |
| *Angyocalyx pynaertii* | MANJOMBE | PAPILLIONACEAE | FR | 509,3 | 11,0 | 29,4 | 1174,5 | 38,0 | 6,6 | 27,5 |
| *Annonidium manii* | MOBEI | ANNONACEAE | FR | 362,5 | 16,6 | 38,2 | 1445,6 | 35,9 | 9,9 | 54,7 |
| *Desplatia dewerei* | LIAMBA | TILIACEAE | FR | 1223,6 | 12,9 | 69,9 | 1914,8 | 63,1 | 6,2 | 25,3 |
| *Dialium zenkeri or pachyphylum* | MBASO | CAESALPINACEAE | FR | 446,8 | 4,6 | 68,3 | 492,6 | 65,7 | 10,7 | 11,3 |
| *Diospyros crassiflora* | LEMBE | EBENACEAE | FR | 523,3 | 3,5 | 18,4 | 644,9 | 71,7 | 4,0 | 7,5 |
| *Duboscia macrocarpa* | NGULUMA | TILIACEAE | FR | 1076,4 | 6,3 | 36,6 | 1043,4 | 108,3 | 0,0 | 4,1 |
| *Dyospiros manii* | MOLOMBO | EBENACEAE | FR | 901,8 | 5,9 | 13,9 | 1586,1 | 96,3 | 0,0 | 14,7 |
| *Gambeya lacourtiana* | BAMBU | SAPOTACEAE | FR | 373,5 | 5,7 | 34,6 | 582,6 | 25,7 | 11,7 | 12,4 |
| *Haumania danckelmaniana* | DJELE | MARANTHACEAE | FR | 331,5 | 16,7 | 66,5 | 1023,7 | 130,5 | 4,3 | 20,9 |
| *Hexabolus crispiflorus* | POTA | ANNONACEAE | FR | 763,1 | 19,5 | 57,9 | 1487,4 | 62,7 | 7,8 | 10,4 |
| *Klainnodoxa gabonensis* | BOKOKO | IRVINGIACEAE | FR | 3290,9 | 9,9 | 21,3 | 868,3 | 221,9 | 0,0 | 10,8 |
| *Myrianthus arboreus* | NGATA | MORACEAE | FR | 1777,0 | 12,5 | 37,0 | 2521,7 | 23,8 | 227,5 | 115,3 |
| *Nauclea d??* | MOSSE TI NGU | RUBIACEAE | FR | 2500,6 | 5,6 | 29,6 | 1781,8 | 20,0 | 2,1 | 9,5 |
| *Pancovia laurentii* | INGOYO | SAPINDACEAE | FR | 538,6 | 13,4 | 16,3 | 1572,2 | 40,1 | 29,1 | 18,0 |
| *Strombosia postulata* | EMBONGO | OLACACEAE | FR | 1577,9 | 16,0 | 56,5 | 980,5 | 246,6 | 14,8 | 18,1 |
| *Tetrapleura tetraptera* | EKOMBOLO | MIMOSACEAE | FR | 1477,4 | 6,0 | 33,5 | 1438,6 | 211,6 |  | 31,0 |
| *Vitex doniana* | MONGWENGWEA | VERBENACEAE | FR | 855,4 | 11,7 | 41,9 | 1508,7 | 183,7 | 25,0 | 24,3 |
| *Cubitermes sp.* | KUSSU | TERMITINAE | IN | 55,6 | 23,9 | 8053,5 | 750,9 | 41,7 | 1719,4 | 58,4 |
| *Dioscorea sp.* | EKULE | DIOSCORIACEAE | LV | 6420,8 | 10,9 | 113,6 | 2878,9 | 1024,2 |  | 32,7 |
| *Tomadersia sp.* | INGUKA | ACANTHACEAE | LV | 4833,4 | 25,6 | 117,4 | 4247,7 | 456,4 |  | 63,6 |
| *Whitefieldia elongata* | INDOLU | ACANTHACEAE | LV | 15560,3 | 14,0 | 203,8 | 10891,5 | 211,9 | 16,2 | 85,0 |
| *Gilbertiodendron dewevrei* | MALAPA | CAESALPINACEAE | SEED | 1197,7 | 8,6 | 32,6 | 1059,3 | 6,7 | 2,1 | 9,3 |
| *(Dialium pachyphylum)* | PURU_MBASO | CAESALPINACEAE | SEED-DUNG | 3149,0 | 27,2 | 79,3 | 3399,3 | 321,3 | 0,2 | 41,4 |
| *Aframonum sp.* | INJOMBO | ZINGIBERACEAE | ST | 1103,2 | 11,3 | 87,3 | 6025,1 | 239,6 | 66,0 | 233,5 |
| *Aframonum subsericium* | INJOKOKO | ZINGIBERACEAE | ST | 1466,3 | 8,2 | 81,6 | 2519,1 | 629,0 | 75,7 | 106,0 |
| *Eichornia crassipes* | CONGWASSIKA | PONTEDERIACEAE | ST | 5818,2 | 5,7 | 0,0 | 2718,1 | 826,1 | 1721,5 | 57,8 |
| *Haumania danckelmaniana* | DJELE | MARANTHACEAE | ST | 624,1 | 30,6 | 2269,5 | 2866,0 | 291,4 | 1,1 | 84,0 |
| *Palisota ambigua* | DOTO | COMMELINACEAE | ST | 4015,3 | 8,7 | 8,2 | 1090,3 | 627,2 | 24,5 | 15,7 |
| *Palisota brachithyrsa or thollonii* | MANGABO | COMMELINACEAE | ST | 4225,2 | 7,0 | 26,8 | 2606,0 | 418,7 | 24,8 | 14,8 |
| *Scleria spp.* | KYEYE | CYPERACEAE | ST | 1617,3 | 2,5 | 221,3 | 856,0 | 830,3 | 7,7 | 32,1 |
| *Angyocalyx pynaertii* | MANJOMBE | PAPILLIONACEAE | YLV | 1229,1 | 13,6 | 71,9 | 1518,8 | 218,0 | 0,3 | 50,2 |
| *Celtis mildbraedii* | NGOMBE | ULMACEAE | YLV | 4381,6 | 11,3 | 99,7 | 2740,1 | 707,7 | 14,2 | 5,9 |
| *Dialium zenkeri or pachyphylum* | MBASO | CAESALPINACEAE | YLV | 1167,8 | 15,2 | 44,4 | 794,7 | 92,1 | 0,8 | 19,6 |
| *Duboscia macrocarpa* | NGULUMA | TILIACEAE | YLV | 8106,4 | 17,5 | 322,4 | 3132,6 | 1213,4 | 4,3 | 19,0 |
| *Gilbertiodendron dewevrei* | MALAPA | CAESALPINACEAE | YLV | 1408,1 | 18,2 | 46,3 | 1966,4 | 15,4 | 0,0 | 19,6 |
| *Milletia sp.* | INGANDA | PAPILLIONACEAE | YLV | 2691,2 | 14,8 | 97,5 | 1976,3 | 172,4 | 43,7 | 64,6 |
|  |  |  |  |  |  |  |  |  |  |  |
